# Preoperative computed tomography evaluation of the paranasal sinuses: what should the physician know? - pictorial essay

**DOI:** 10.1590/0100-3984.2017.0082

**Published:** 2019

**Authors:** Bruno Niemeyer de Freitas Ribeiro, Bernardo Carvalho Muniz, Edson Marchiori

**Affiliations:** 1 Instituto Estadual do Cérebro Paulo Niemeyer - Departamento de Radiologia, Rio de Janeiro, RJ, Brazil.; 2 Universidade Federal do Rio de Janeiro (UFRJ), Rio de Janeiro, RJ, Brazil.

**Keywords:** Tomography, X-ray computed, Paranasal sinuses, Rhinitis, Sinusitis, Tomografia computadorizada, Seios paranasais, Rinite, Sinusite

## Abstract

The introduction of functional endoscopic sinus surgery in the 1980s brought about a drastic change in the treatment of patients with rhinosinusitis, improving quality of life through the removal of pathological processes or anatomical variations that obstruct the drainage pathways of the paranasal sinuses. However, despite the routine use of computed tomography in the anatomical evaluation of the paranasal sinuses, most radiological reports still do not provide sufficient information to guide the surgical planning. The objective of this pictorial essay was to demonstrate, through computed tomography, the main anatomical variations of the paranasal sinuses, the recognition of which is fundamental for preoperative planning, in order to avoid treatment failure and iatrogenic complications.

## Abstract (pt)

A introdução da cirurgia endoscópica sinusal funcional na década de 80 proporcionou uma mudança drástica no tratamento de pacientes com rinossinusite, melhorando a qualidade de vida mediante a retirada de processos patológicos ou variações anatômicas que provocam obstrução nas vias de drenagem dos seios paranasais. Porém, apesar do uso rotineiro da tomografia computadorizada na avaliação anatômica dos seios paranasais, a maioria dos laudos radiológicos ainda carece de informações que orientem o planejamento cirúrgico. O objetivo deste ensaio iconográfico é demonstrar, por meio de tomografia computadorizada, as principais variações anatômicas dos seios paranasais, cujo reconhecimento é fundamental para o planejamento pré-operatório, a fim de evitar falhas terapêuticas e iatrogenias.

## INTRODUCTION

The introduction of functional endoscopic sinus surgery in the 1980s brought about a drastic change in the treatment of patients with recurrent or refractory rhinosinusitis, alleviating symptoms and improving quality of life in more than 75% of patients^(^^1^^,^^2^^)^. The intention of the surgery is to remove pathological processes or anatomical variations that obstruct the drainage pathways of the paranasal sinuses, the main targets being the ostiomeatal complex and the frontal recess, and may often include uncinectomy and maxillary antrostomy, as well as turbinectomy, turbinoplasty, ethmoidectomy, and frontal sinusotomy^(^^1^^)^.

The risk of complications in functional endoscopic sinus surgery is rare, such complications occurring in only 0.36-1.30% of cases and being more common among patients that have previously undergone the procedure, as well as among those in whom the surgical intervention is more extensive^(^^1^^,^^3^^,^^4^^)^. The most serious complications are lesions of the anterior ethmoidal artery, optic nerve, or nasolacrimal duct, as well as cerebrospinal fluid leaks^(^^1^^,^^3^^,^^4^^)^.

Recent advances in computed tomography (CT) and magnetic resonance imaging techniques have increased the importance of imaging studies in the evaluation of diseases that affect the head and neck^(^^5^^-^^10^^)^. Although CT scans are used routinely in anatomical evaluations of the paranasal sinuses, a recent study showed that 75% of radiological reports add little value in terms of informing therapeutic decisions^(^^1^^,^^11^^)^. The aim of this study was to demonstrate, using CT, the main anatomical variations that can affect the surgical planning.

## EVALUATION OF THE UNCINATE PROCESS

The uncinate process is a superior extension of the lateral nasal wall and plays an important role, as an anatomical landmark, in guiding drainage of the frontal recess. According to Landsberg and Friedman^(^^12^^)^, variations in the insertion of the uncinate process are classified as follows:

- type 1 - insertion into the lamina papyracea (Figure 1);
  **Figure 1:**
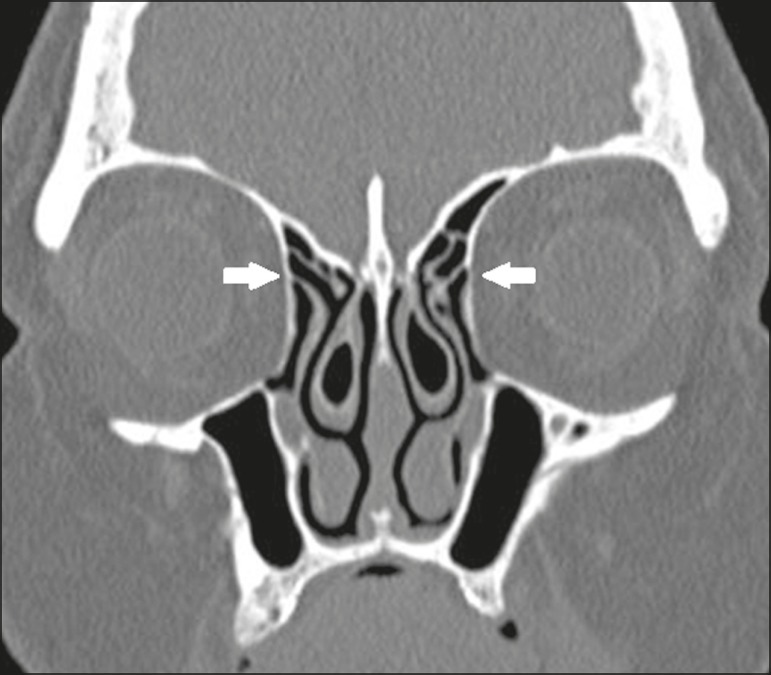
Coronal CT slice, with bone window settings, showing the insertion of uncinate processes into the lamina papyracea (arrows), characteristic of a Landsberg and Friedman type 1 insertion.
- type 2 - insertion into the posterior wall of the agger nasi cell;
- type 3 - insertion into the lamina papyracea and at the junction of the middle turbinate with the cribriform plate (Figure 2);
  **Figure 2:**
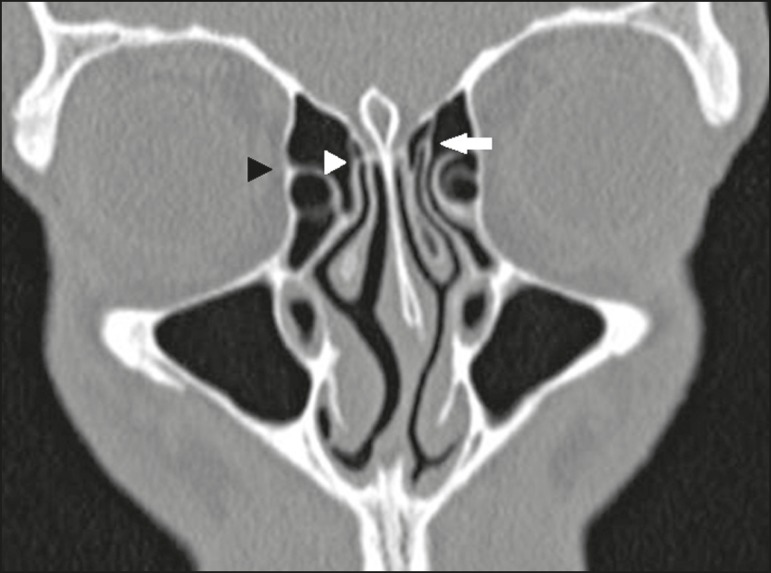
Coronal CT slice, with bone window settings, showing the insertion of the right uncinate process into the lamina papyracea (black arrowhead), and at the junction of the middle turbinate with the cribriform plate (white arrowhead), characteristic of a Landsberg and Friedman type 3 insertion, as well as the insertion of the left uncinate process at the skull base (white arrow), characteristic of a Landsberg and Friedman type 5 insertion.
- type 4 - insertion at the junction of the middle turbinate with the cribriform plate (Figure 3);
  **Figure 3:**
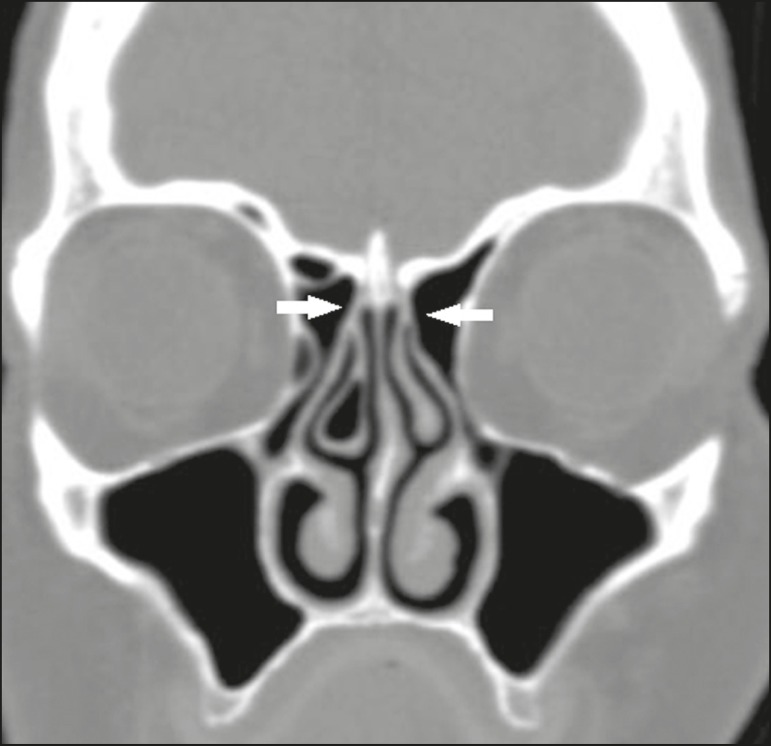
Coronal CT slice, with bone window settings, showing the insertion of the uncinate processes at the junction of the middle turbinate with the cribriform plates (arrows), characteristic of a Landsberg and Friedman type 4 insertion.
- type 5 - insertion into the base of the skull (Figure 2);
- type 6 - insertion into the middle turbinate (Figure 4).
  **Figure 4:**
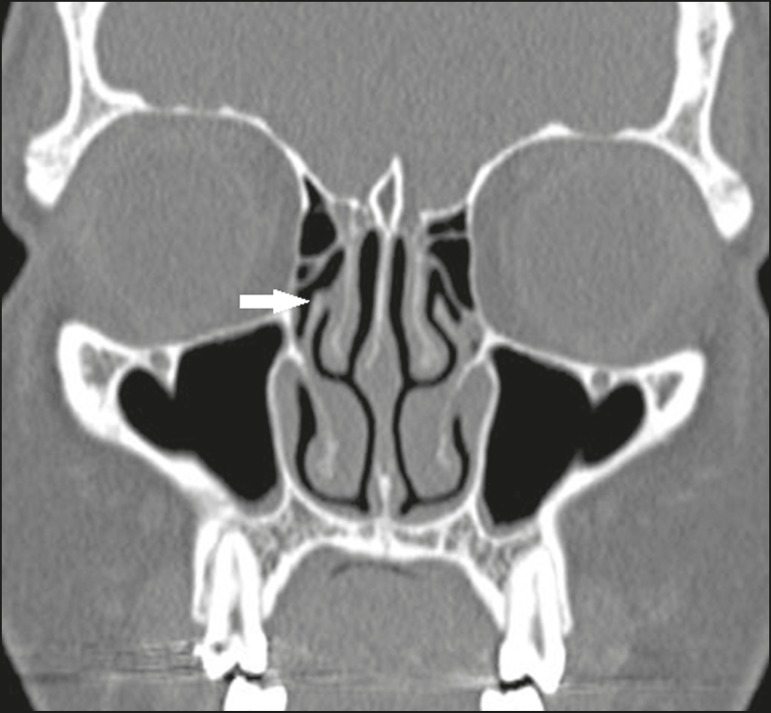
Coronal CT slice, with bone window settings, showing the insertion of the right uncinate process into the middle turbinate (arrow), characteristic of a Landsberg and Friedman type 6 insertion.

Inadvertent manipulation in insertion types 1 or 3 may provoke lesion in the lamina papyracea, with herniation of the orbital contents and orbital hematoma, whereas inadvertent manipulation of skull base insertions (types 3, 4, and 5) can cause cerebrospinal fluid leaks^(^^1^^)^.

**Figure 5:**
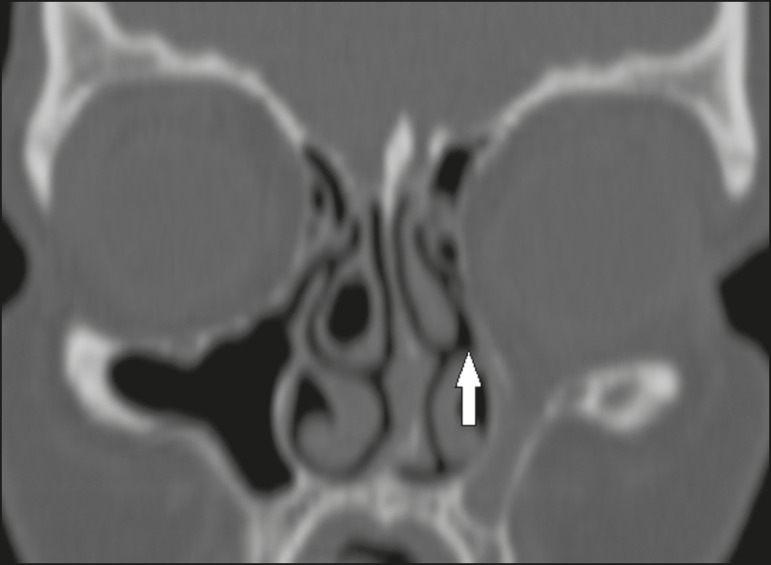
Coronal CT slice, with bone window settings, showing atelectasis of the left maxillary sinus, causing lateral deviation of the uncinate process and apposition to the medial orbital wall (arrow).

It is important to specify when there is under-pneumatization or atelectasis of the maxillary sinus, because either can cause lateral deviation of the uncinate process and apposition to the medial orbital wall^(^^1^^)^ (Figure 5).

## DEPTH OF THE OLFACTORY FOSSA

**Figure 6:**
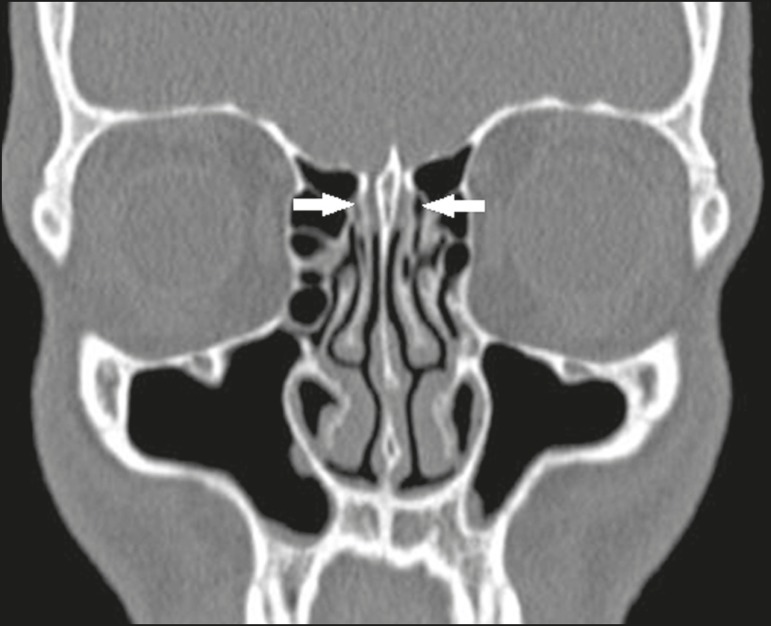
Coronal CT slice, with bone window settings, showing symmetry in the depth and inclination of the olfactory fossae (arrows), each measuring approximately 7.5 mm in depth and therefore classified as Keros type 3.

The Keros classification uses coronal CT reconstruction to evaluate the depth of the olfactory fossa in relation to the ethmoid roof, taking as a reference the length of the lateral lamella of the cribriform plate (Figure 6). Greater depth of the olfactory fossa translates to a greater chance that it will be injured during surgery, especially during turbinectomy or ethmoidectomy, consequently risking cerebrospinal fluid leaks and loss of the sense of smell^(^^1^^,^^12^^,^^13^^)^.

The Keros classification comprises the following types:

- type 1 - olfactory fossa depth ≤ 3 mm;
- type 2 - olfactory fossa depth > 3 mm and ≤ 7 mm (the most common type);
- type 3 - olfactory fossa depth > 7 mm.

**Figure 7:**
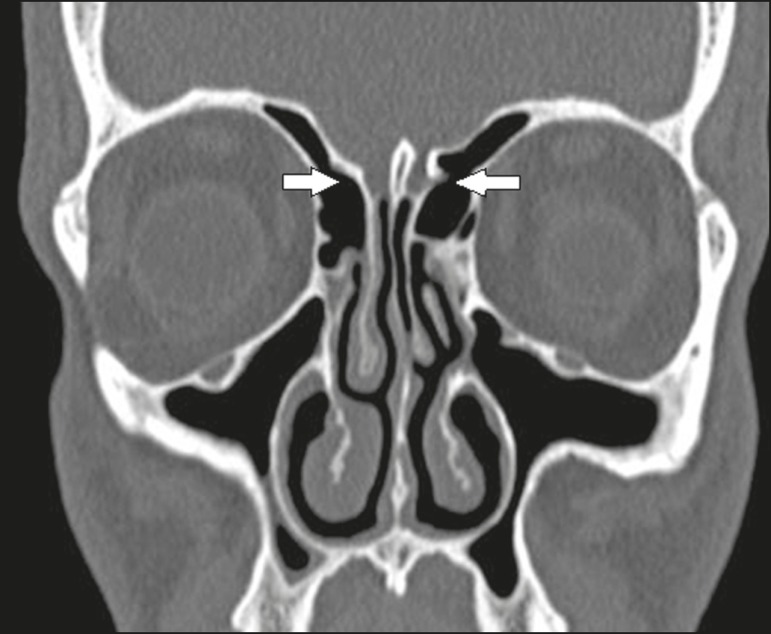
Coronal CT slice, with bone window settings, showing asymmetry of the olfactory fossae (arrows).

It is also important to note asymmetries in the inclination of the olfactory fossae^(^^1^^,^^12^^,^^13^^)^ (Figure 7).

## ANTERIOR ETHMOIDAL ARTERY

The anterior ethmoidal artery is responsible for irrigating the anterior ethmoid cells, the frontal sinus, the anterior third of the nasal septum, and the lateral nasal wall. It penetrates the olfactory fossa from the lateral lamella of the cribriform plate, through the so-called anterior ethmoidal groove, the most fragile portion of the anterior skull base.

**Figure 8:**
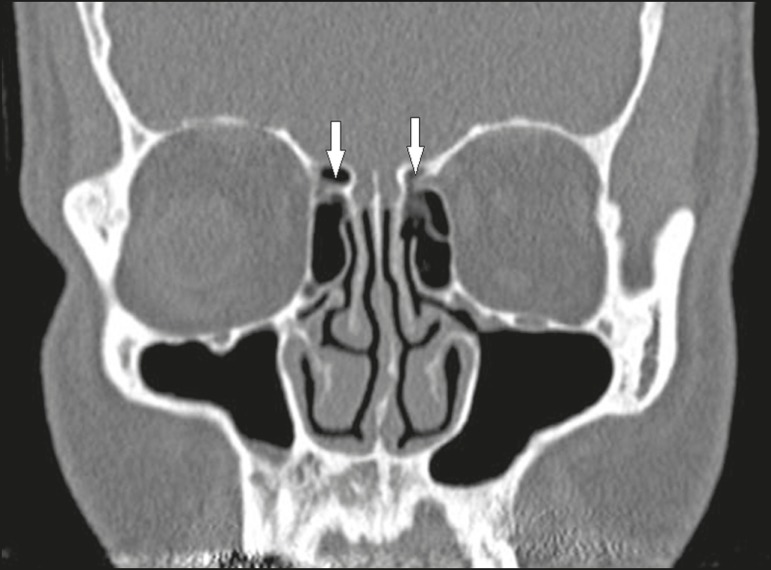
Sagittal CT slice, with bone window settings, showing the anterior ethmoidal arteries (arrows). In this case, there were no supraorbital cells.

The main anatomical sites at which the anterior ethmoidal artery can be found are the notch in the medial wall of the orbit (anterior ethmoidal foramen) and the anterior ethmoidal groove in the lateral wall of the olfactory fossae (Figure 8); the exact location of the artery can be best evaluated on a coronal CT scan^(^^1^^,^^14^^)^. Knowing the exact location of the anterior ethmoidal artery helps avoid intraoperative bleeding, which often occurs when there are supraorbital ethmoid cells or when the artery is exposed^(^^1^^,^^14^^)^.

## DEHISCENCE OF THE LAMINA PAPYRACEA

**Figure 9:**
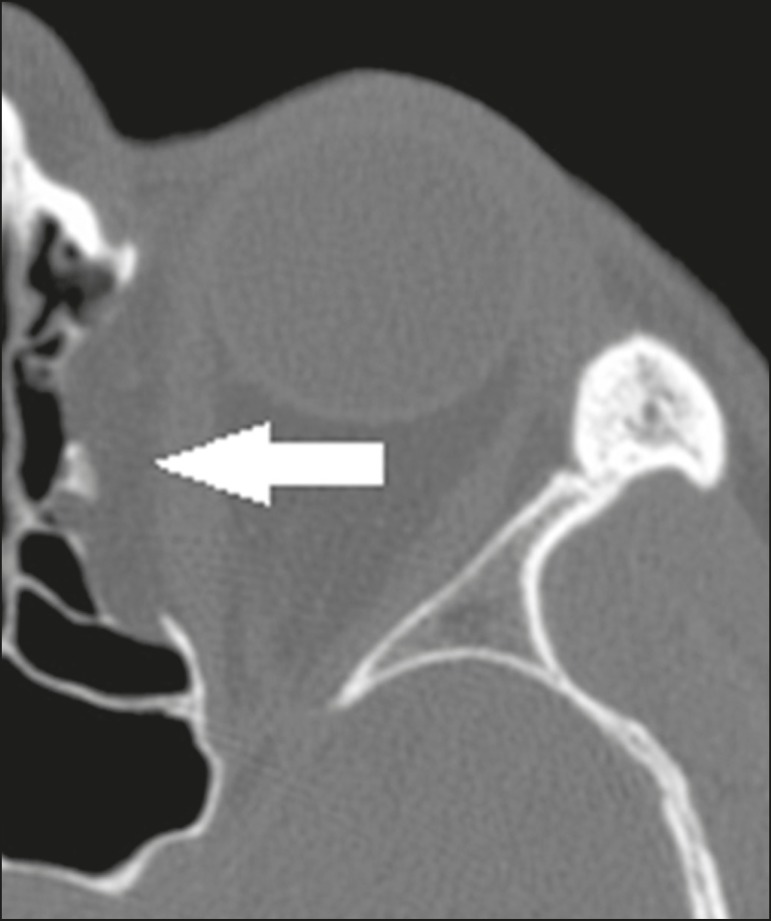
Axial CT slice, with bone window settings, showing dehiscence of the lamina papyracea (arrow).

The lamina papyracea is a thin bony layer of the ethmoid that forms the medial orbital wall. On CT, it is best evaluated in the coronal and axial planes. When dehiscence occurs, the bone margins are displaced to the ethmoid cells medially, with the insinuation of orbital fat and occasionally even portions of the medial rectus muscle (Figure 9). This alteration can be confused with septation of the ethmoid sinus during the surgical procedure and can increase the risk of intraoperative penetration^(^^1^^)^.

## INFRAORBITAL ETHMOID CELL (HALLER CELL)

**Figure 10:**
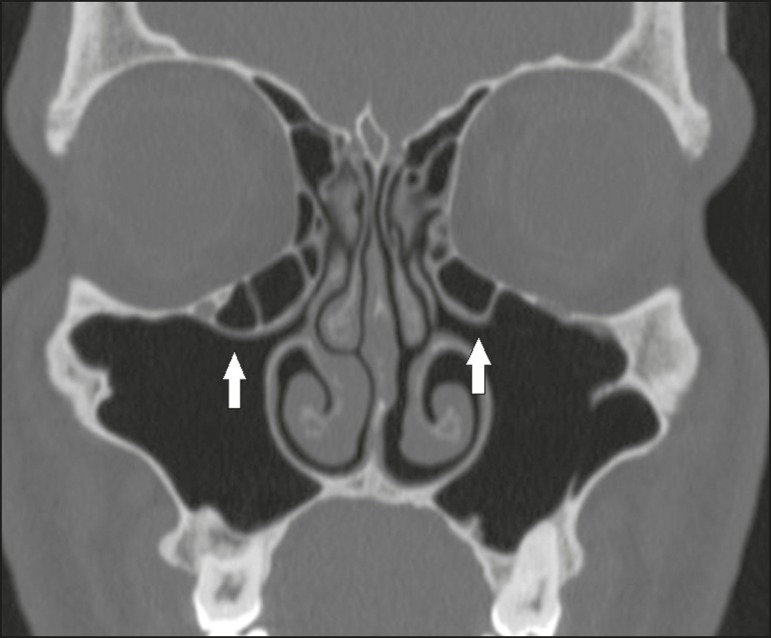
Coronal CT slice showing Haller cells, bilaterally (arrows), that are more clearly visible on the right, insinuation into the maxillary sinuses, reducing the amplitude of the ethmoid infundibula.

Infraorbital ethmoid cells (Haller cells) are ethmoid cells that are pneumatized inferiorly to the orbital floor, and extend from the ethmoidal labyrinth to the interior of the maxillary sinus (Figure 10), which can cause obstruction and predisposition to sinus diseases^(^^1^^,^^12^^,^^15^^)^. Inadvertent manipulation can damage the lamina papyracea^(^^1^^)^.

## FRONTAL CELLS (KUHN CELLS)

Found in 20-30% of patients, frontal ethmoid cells, also known as Kuhn cells, are closely related to agger nasi cells and are divided into four types^(^^1^^,^^12^^,^^16^^)^:

- type 1 - single cell, located above the agger nasi cell and below the frontal sinus floor;
- type 2 - two or more anterior ethmoid cells that are pneumatized above the agger nasi cell and can extend into the frontal sinus;
- type 3 - single anterior ethmoid cell that, due to its large volume, is pneumatized above the agger nasi cell and extends into the frontal sinus (Figure 11);
  **Figure 11:**
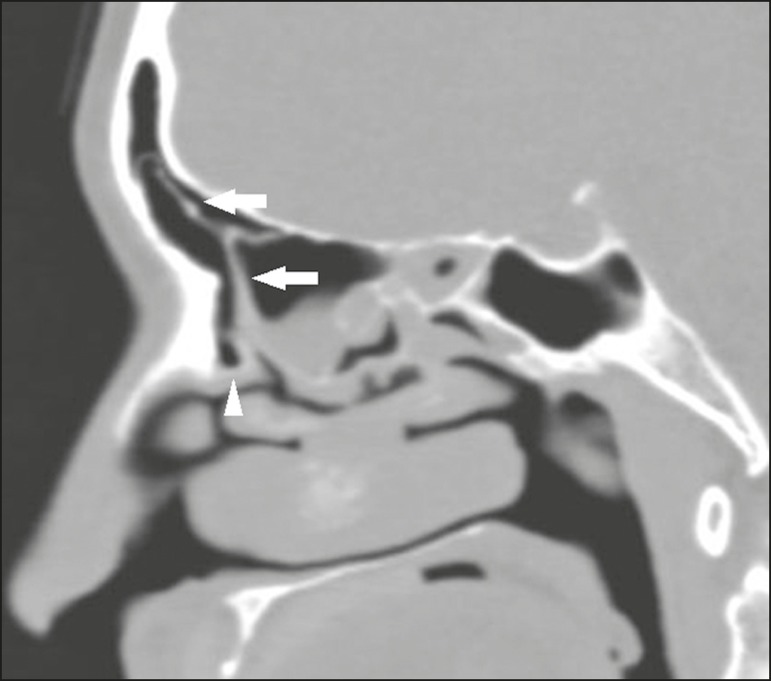
Sagittal CT slice, with bone window settings, showing a large single anterior ethmoid cell (arrows), extending into the frontal sinus and located above the agger nasi cell (arrowhead).
- type 4 - a rare, isolated cell, located within the frontal sinus, seen only in 2.4% of individuals^(^^11^^)^.

Diseases of the frontal sinus are more prevalent in patients who have type III and IV frontal cells than in those who have no frontal cells^(^^1^^)^.

## FRONTAL SINUS CELLS

**Figure 12:**
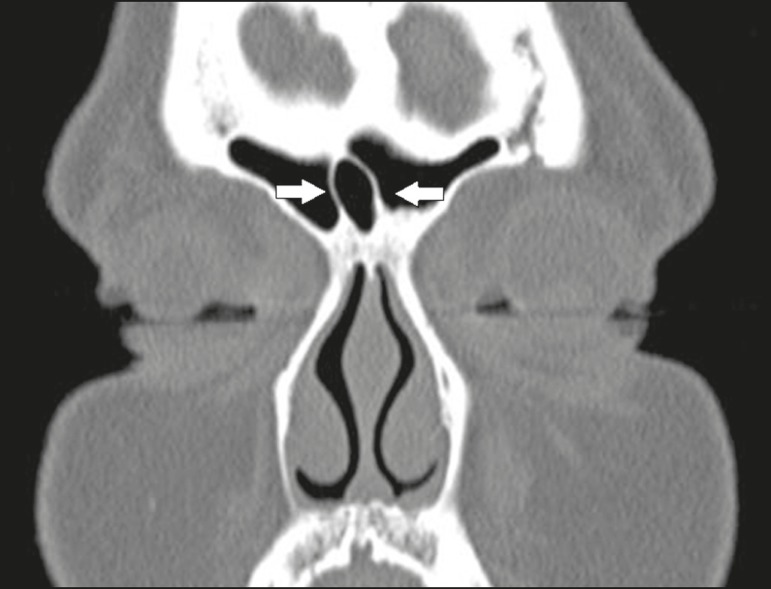
Coronal CT slice, with bone window settings, showing the frontal sinus cell (arrows).

Pneumatization of the bony lamella (septum) between the frontal sinuses, or a frontal sinus cell (Figure 12), can create confusion during frontal sinus surgery. Such pneumatization can also predispose to the formation of mucoceles.

## SPHENOETHMOIDAL CELLS (ONODI CELLS)

**Figure 13:**
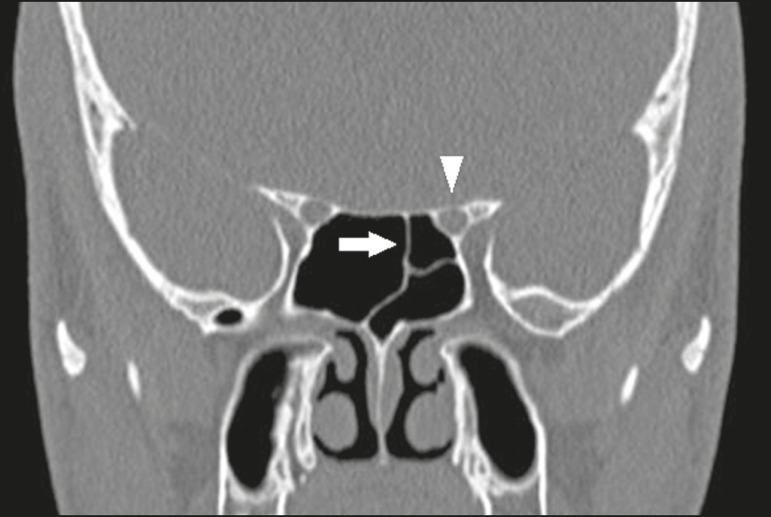
Coronal CT slice, with bone window settings, showing the left sphenoethmoidal cell (arrow). Note the close proximity to the left optic nerve (arrowhead).

Sphenoethmoidal cells, also known as Onodi cells, are posterior ethmoid cells that migrate to the superolateral aspect of the sphenoid sinus, in close proximity to the optic nerve; on CT, they are best evaluated in the coronal plane^(^^1^^,^^16^^)^, as shown in Figure 13. Inadvertent manipulation, especially during posterior ethmoidectomy, can damage the corresponding optic nerve^(^^1^^,^^12^^,^^16^^)^.

## PNEUMATIZATION OF THE SPHENOID SINUS

**Figure 14:**
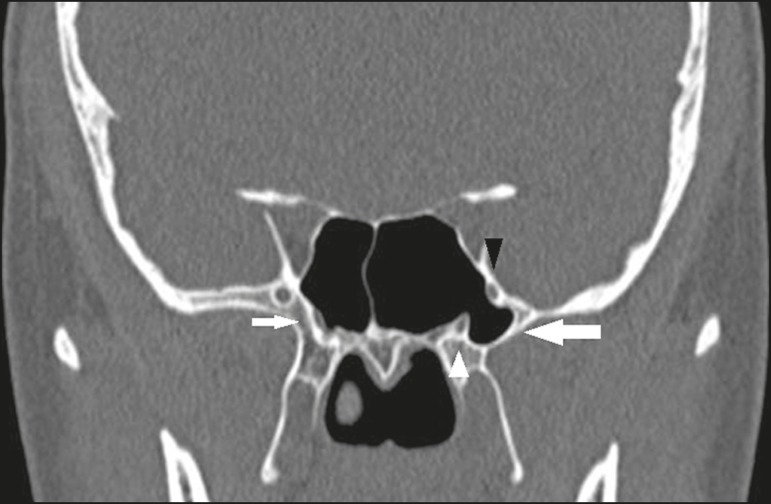
Coronal CT slice, with bone window settings, showing the left pterygoid recess (thick white arrow) in close proximity to the foramen rotundum (black arrowhead) and the vidian canal (white arrowhead). Note the absence of pneumatization of the right pterygoid recess (thin white arrow).

**Figure 15:**
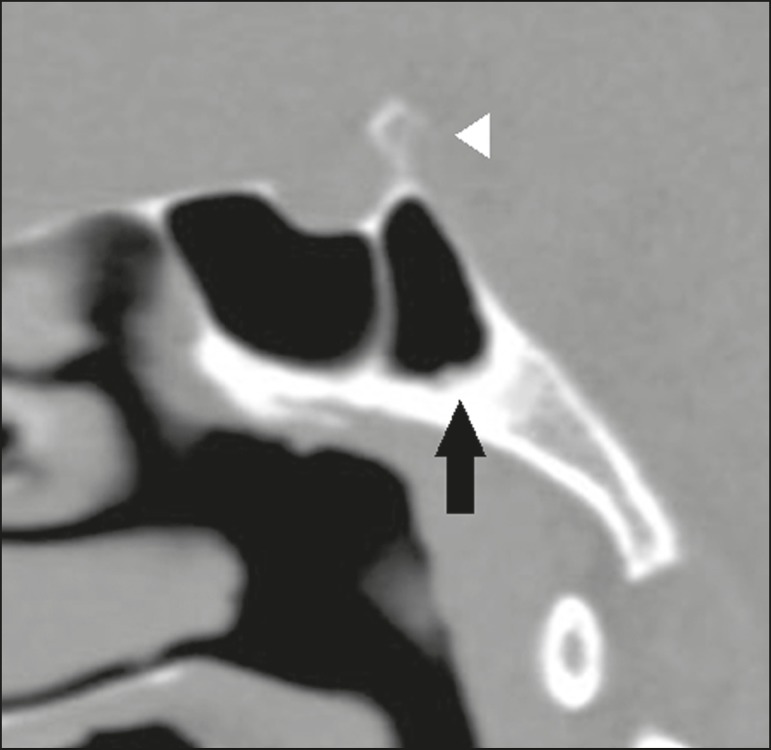
Sagittal CT slice, with bone window settings, showing pneumatization of the sphenoid sinus extending inferiorly and posteriorly to the sella turcica (black arrow), classified as the sellar variant. Dorsal view of the sella turcica (white arrowhead).

Pterygoid recesses are created by pneumatization of the lateral recesses of the sphenoid sinuses (Figure 14), predisposing to lesions in the foramen rotundum and vidian canal if there is inadvertent surgical manipulation^(^^1^^)^. It is also important to evaluate pneumatization of the sphenoid sinuses in relation to the sella turcica and the clivus, which can be achieved by performing sagittal CT reconstructions, the sellar variant (pneumatization extending inferiorly and posteriorly to the sella turcica), as depicted in Figure 15, increasing the risk of perforation and inadvertent intracranial access^(^^1^^)^.

It is also important to identify dehiscence of the carotid canal, as well as its insinuation into the sphenoid sinuses^(^^1^^)^. Adherence of the intersphenoid septum to the carotid canal should be noted^(^^1^^)^.

## CHANGES IN NASAL TURBINATES

**Figure 16:**
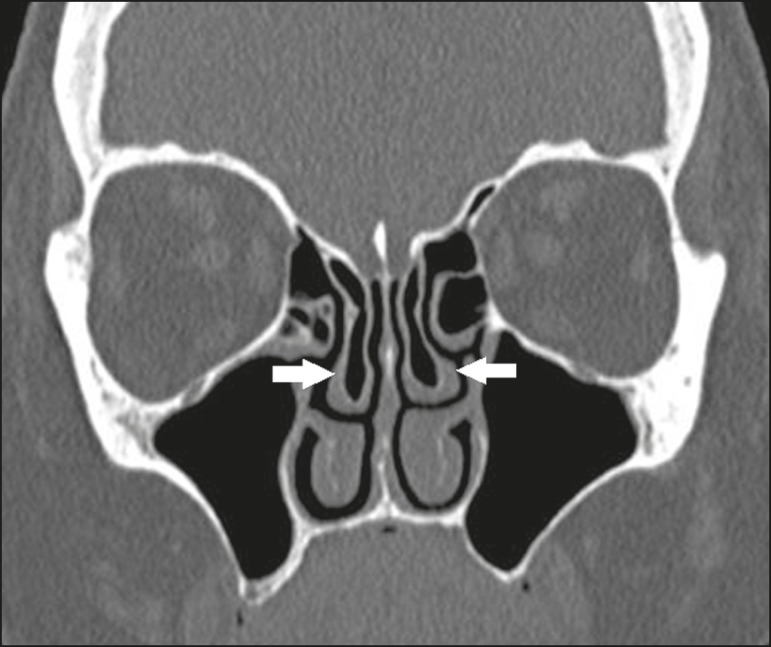
Coronal CT slice, with bone window settings, showing pneumatization of the lamellar and bulbous portions of the middle turbinate (arrows).

**Figure 17:**
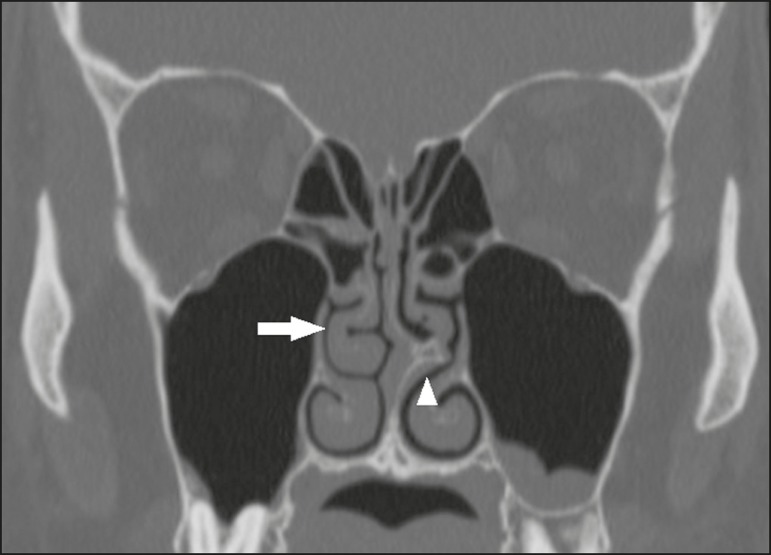
Coronal CT slice, with bone window settings, showing a paradoxical right middle turbinate (arrow). Also note the pronounced bone spur on the left side of the nasal septum (arrowhead).

Pneumatized or paradoxical middle turbinates (Figures 16 and 17, respectively) can narrow the middle meatus and cause lateral deviation of the uncinate process, with a consequent reduction in the amplitude of the ethmoid infundibulum^(^^1^^)^.

## CONCLUSION

CT is an extremely useful tool for the evaluation of the sinuses and their anatomical variations, providing information that is essential for diagnosis and therapeutic planning. Radiologists should be aware of the indications for CT, in order to improve the clinical decision-making process.
